# Supplementary material for: COVID-19 Vaccines Status, Acceptance and Hesitancy among Maintenance Hemodialysis Patients: A Cross-Sectional Study and the Implications for Pakistan and Beyond
Source: Vaccines (Basel). 2023 Apr 27;11(5):904. doi: 10.3390/vaccines11050904 (PMC10223584; doi:10.3390/vaccines11050904)
Supplement: Supplementary file 1 [file vaccines-11-00904-s001.zip › vaccines-2324513-supplementary.pdf]

Covid-19 vaccines acceptance and hesitancy among maintenance hemodialysis patients in Punjab, Pakistan

Research purpose:

This survey will not take more than 5-10 minutes of your time. All data collected will be used only for research purposes & will be kept confidential. No connection will be possible between your identity and the answers you provide.

<u>Section 1: Demographics and Hemodialysis related information</u>

**Q1**	**Gender**
1	Male
2	Female
**Q2**	**Age (Years)**
1	Below 18
2	18–44
3	45–64
4	65–79
5	Above 80
**Q3**	**Residence**
1	Urban
2	Rural
**Q4**	**Marital status**
1	Single/divorced/widow
2	Married
**Q5**	**Occupation**
1	Student
2	Employed
3	Unemployed
4	Retired
5	Cannot work due to disability
**Q6**	**Household average monthly income?**
1	Less than 30000 PKR

2	31000-60000 PKR
3	More than 60000 PKR
**Q7**	**Education level?**
1	Illiterate
2	Religious education
3	Primary school
4	High school
5	Higher secondary education
6	Diploma
7	Bachelor's degree
8	Postgraduation
**Q8**	**Do you smoke?**
1	Yes
2	No
3	Ex-smokers
**Q9**	**For how many years do you undergoing hemodialysis?**
1	Less than one year
2	More than one - three years
3	More than three years
**Q10**	**Frequency of hemodialysis per week**
1	Once a week
2	Two times a week
3	Three times a day
**Q11**	**Causes of chronic renal failure**
1	Congenital kidney abnormalities
2	Hypertension and diabetes mellitus
3	Nephrolithiasis
4	Analgesic nephropathy
5	Chronic pyelonephritis
6	Diabetes mellitus
7	Glomerulonephritis

8	Hypertension		
9	Hypovolemia		
10	Pregnancy related		
11	Amyloidosis		
12	Schistosomiasis		

Q12 Associated co morbidities

Yes

No

1	Diabetes	Yes	No
2	Hypertension	Yes	No
3	Chronic lung disease	Yes	No
4	ischemic heart disease	Yes	No
5	No chronic illness	Yes	No

Q13 Current medications (You can select multiple options)

1	Erythropoietin	Yes	No
2	Iron supplementation	Yes	No
3	Calcium supplementation	Yes	No
4	Vitamin D	Yes	No
5	Calcimimetics	Yes	No
6	Aluminum hydroxide	Yes	No
7	Antihypertensives drugs	Yes	No
8	Antidiabetic drugs	Yes	No

Q14 Influenza vaccination status

1 Vaccinated

2 Not vaccinated

Q15 Hepatitis B vaccination status

1 Vaccinated

2 Not vaccinated

Q16 Current Hepatitis C status

1 Positive

2 Negative

Q17 Family member get infected with COVID-19

1 Yes

2 No

Q18 Family member died with COVID-19

1 Yes

2 No

Q19 Did you get covid-19 Vaccine?

1 Yes

2 No

Q20 If yes how many doses

1 One

2 One + two

3 One+two+ booster

Section 2: Reasons for vaccines acceptance

(This section only collects information for those patients that have receive at least one COVID-19 vaccine dose)

What were the reasons to get/accept COVID-19 vaccines? (You can select multiple options).

Sr. No.	Reasons of COVID-19 vaccine acceptance	Yes	No
1	I didn't fear possible side effects of the COVID-19 vaccines		
2	The vaccination is for free		
3	The desire to return to normal life as soon as possible		
4	I want to participate fight against COVID-19		
5	I have no doubt about the safety of COVID-19 vaccines		
6	Fear of getting COVID-19		
7	Fear of transmitting COVID-19 to others		
8	I am at high risk of acquiring COVID-19 infection		

9	Government makes it compulsory for everyone to get vaccine		

Willingness to get COVID-19

If you didn't get vaccines yet, are you willing to get it?

1 Yes

2 No

If you are not willing to get COVID-19 what are reasons for it?

Section 3: Reasons for vaccines hesitancy

(This section only collects information for those patients that didn't receive or willing to receive COVID-19 vaccine)

What were the reasons not to get/accept COVID-19 vaccines? (You can select multiple options).

Sr. No.	Reasons of COVID-19 vaccine hesitancy	Yes	No
1	I am concerned about the side effects of the COVID19 vaccine		
2	I don't feel comfortable with vaccines in general		
3	I am concerned that the vaccine will not work		
4	I don't think I need it		
5	I heard or read news that COVID19 vaccine is dangerous		
6	I have had a bad prior reaction to another vaccine		
7	I already had COVID19		
8	I believe it is better to get natural infection than a vaccine		
9	I don't believe COVID19 is a real problem		
10	The vaccine is a conspiracy		
11	If lots of other people get the vaccine, I won't need it		
12	Others		
